# Stretching the Function of Innate Immune Cells

**DOI:** 10.3389/fimmu.2021.767319

**Published:** 2021-11-02

**Authors:** Erica M. Orsini, Apostolos Perelas, Brian D. Southern, Lisa M. Grove, Mitchell A. Olman, Rachel G. Scheraga

**Affiliations:** ^1^ Respiratory Institute, Cleveland Clinic, Cleveland, OH, United States; ^2^ Department of Pulmonary and Critical Care, Virginia Commonwealth University, Richmond, VA, United States; ^3^ Department of Inflammation and Immunity, Lerner Research Institute, Cleveland Clinic, Cleveland, OH, United States

**Keywords:** innate immunity, mechanotranduction, macrophage, neutrophil, integrins, TRPV4, Piezo1

## Abstract

The importance of innate immune cells to sense and respond to their physical environment is becoming increasingly recognized. Innate immune cells (e.g. macrophages and neutrophils) are able to receive mechanical signals through several mechanisms. In this review, we discuss the role of mechanosensitive ion channels, such as Piezo1 and transient receptor potential vanilloid 4 (TRPV4), and cell adhesion molecules, such as integrins, selectins, and cadherins in biology and human disease. Furthermore, we explain that these mechanical stimuli activate intracellular signaling pathways, such as MAPK (p38, JNK), YAP/TAZ, EDN1, NF-kB, and HIF-1α, to induce protein conformation changes and modulate gene expression to drive cellular function. Understanding the mechanisms by which immune cells interpret mechanosensitive information presents potential targets to treat human disease. Important areas of future study in this area include autoimmune, allergic, infectious, and malignant conditions.

## Introduction

The ability of innate immune cells to sense and respond to the physical environment is critical for their function. Through the study of mechano-immunology, it is now increasing understood that mechanical cues are just as important as biochemical cues for determining immune cell activation (1). Immune cells encounter a wide range of environmental conditions while performing immune functions. For example, different tissues have vastly different mechanical properties with bone (10-20x10^6^ kPa) significantly stiffer than lung (1 kPa) (2, 3). Pathologic condition may cause the stiffness of tissue to change, for example, pulmonary inflammation/fibrosis causes the stiffness of the lung to increase 6-20-fold from 1 kPa to 6-20 kPa (3). During both physiological and pathological states, cells receive mechanical input from the biophysical properties of the extracellular environment, including matrix stiffness, stretch, shear force, elasticity, and shape (4).

Through mechanotransduction, cells translate force into cellular messages that induce protein conformation changes, activate intracellular signaling pathways, and modulate gene expression to drive cellular function (1, 5, 6). For example, depending on the stiffness of the *in vitro* environment, macrophages can develop into the different classically described macrophage phenotypes, pro-inflammatory (“M1-like”) or pro-healing (“M2-like”), however direct extrapolation of the *in vitro* macrophage phenotypes to *in vivo* function is problematic (7, 8). Spatial confinement has also been shown to influence macrophage phenotype with macrophages confined by crowding having less expression of pro-inflammatory cytokines (IL-6, IL-1β) in response to lipopolysaccharide (LPS) in a manner that correlates with impaired actin polymerization and reduced nuclear translocation of myocardin-related transcription factor A (MRTF-A) (9). Similarly in neutrophils, mechanical deformations generated through optical stretching have been shown to influence cytokine secretion (10). Mechanotransduction is critical in non-immune cells as well, where mechanosensitive mechanisms regulate many cell functions including regulation of blood pressure and myotube and bone formation (11–13).

Ion channels, such as Piezo1 and Transient receptor potential cation channel subfamily V member 4 (TRPV4), and cell adhesion molecules (CAMs), such as integrins, selectins, and cadherins, have been shown to play a role in mechanotransduction in innate immune cells (5, 14). In this review, we will explore what is known about the role of ion channels and adhesion molecules in controlling macrophage and neutrophil function. Given the brevity and focus of this review, we acknowledge that we could not include all published work in this area. For further inquiry on this topic, we direct you to comprehensive reviews in this area (1, 5, 15).

## Ion Channels

Cation channels are membrane proteins containing a cation-permeable pore which allow for the passage of cations (including calcium Ca^2+^, magnesium Mg^2+^, potassium K^+^, and sodium Na^2+^) along their electrochemical gradients. Ca^2+^ plays a critical role in innate immune cell signaling. For example, in macrophages, Ca^2+^ has been shown to be an important second messenger in Tumor Necrosis Factor-α (TNF-α) secretion and phagocytosis (16, 17). In neutrophils, the influx of Ca^2+^ allows for degranulation, NADPH oxidase activation, and the generation of reactive oxygen species (18, 19). Ion channels, which control influx of Ca^2+^, have a critical role in cellular mechanotransduction. Mechanosensitive ion channels are ubiquitously expressed in cells and tissues, and recently have been recognized for their important role in immune function. Mechanosensitive mechanisms were initially discovered in neurons, which has now led to exploration of their function in numerous cell types (20). The importance of ion channels in mechanobiology has been recently highlighted by the awarding of the Nobel Prize in Medicine to David Julius and Ardem Patapoutian for their work examining the role of TRP and Piezo channels in sensory function (21). This review will focus on 2 of the best studied mechanosensitive channels for their role in inflammation, Piezo1 and TRPV4.

## Piezo1

The Piezo channels, which include Piezo 1 and 2, are mechanically gated ion channels activated directly (e.g. mechanical stretch, chemical stimulus) or indirectly (e.g. through other channel or force generated downstream signals) (22). In addition to its role in immune function, Piezo1 has been shown to have a role in blood pressure regulation and myoblast fusion during skeletal muscle formation, while Piezo2 regulates sensation of light touch and proprioception (11, 12, 23, 24). Piezo1, Piezo2, and TRPV4 act as key force sensors during bone development and osteoblast differentiation (13, 25). Direct activation of Piezo1 occurs through mechanical stretch of the lipid bilayer which permits the flow of Ca^2+^ across the membrane (26). The mechanogating function of the Piezo1 channel is achieved through a lever-like mechanism which involves both the intracellular and transmembrane domains (27). In the absence of mechanical stimuli, Piezo1 can be activated by the small molecule, Yoda1 (28). During myotube formation, inward translocation of phosphatidylserine (PS) from the phospholipid bilayer of myoblasts is an important mechanism for Ca^2+^ influx *via* Piezo1, which leads to actomyosin assemby, although it is unknown if inward translocation of PS is an important mechanism of Piezo1 activation in myeloid cells (12). There is evidence of Piezo1 activation by force transduction *via* “inside-out” activation through interaction with intracellular cytoskeletal filaments (29). Traction force generated by the cytoskeletal contractile protein Myosin II by Myosin Light Chain Kinase (MLCK) mediates Ca^2+^ influx *via* Piezo1 (30).

Once Piezo1 is activated by mechanical stimuli (pressure or stretch), the Piezo1 signal integrates with that of chemical pro-inflammatory signals to regulate macrophage function. In response to cyclical hydrostatic pressure in murine bone marrow derived macrophages (BMDM), Piezo1 mediates Ca^2+^ influx leading to activator protein-1 (AP-1) activation, production of endothelin-1 (EDN1), and stabilization of hypoxia inducible factor 1α (HIF1α) to modulate important genes that lead to the production of pro-inflammatory mediators, such as IL-6, TNF-α, chemokine ligand 2 (CXCL2), and prostaglandin E2 (31). Stiffer substrates (~kPa-GPa, e.g. tissue culture plastic or glass) lead to increased macrophage bacterial clearance and production of “M1-like” pro-inflammatory cytokines, such as IL-6 and TNF-α, and suppression of “M2-like” pro-healing markers, such as arginase 1 (ARG1) (32). BMDM cultured on stiffer substrates also have increased expression of Piezo1 and enhanced Ca^2+^ influx, suggesting a possibility of a positive feedback loop of matrix stiffness on Piezo1-mediated macrophage function (32).

In addition to mechanical stimuli, Piezo1 has been shown to influence macrophage polarization in response to stimulation by IFN-γ/LPS. Toll-like receptor 4 (TLR4) has been shown to mediate Ca^2+^ influx *via* interaction with Piezo1 in response to LPS (33). Macrophages from mice with myeloid specific deletion of Piezo1 (Piezo1Δ^LysM^) have increased signal transducer and activator of transcription 6 (STAT6) activation, and decreased nuclear factor kappa-light-chain-enhancer of activated B cells (NF-kB) activation, leading to a pro-healing phenotype after stimulation with IFN-γ/LPS (32). It has been shown that Piezo1Δ^LysM^ mice have impaired clearance of *P. aeruginosa* in the lungs compared with wild-type mice, demonstrating that Piezo1 is required for bacterial clearance in a relevant *in vivo* model (31). However, the opposite was shown in a mouse model of polymicrobial sepsis *via* cecal ligation and puncture (CLP). The CLP model, CD11b+ myeloid cells from Piezo1Δ^LysM^ mice had decreased pro-inflammatory cytokines, enhanced peritoneal bacterial clearance, and less sepsis-related death than their Piezo1^fl/fl^ littermates (34). These conflicting data illustrate the need for further research to understand the precise role of Piezo1 in the innate immune system using multiple complementary model systems.

Studies support the existence of a positive feedback loop between mechanosensitive channels, such as Piezo1, and the cytoskeleton in macrophages (32). Inhibition of actin polymerization reduced Ca^2+^ influx *via* Piezo1 (32). Feedback loops between Piezo1 and the cytoskeleton have also been demonstrated in other cell types. For example, Piezo1 activation is necessary for orientation of vascular endothelial cells during embryonic development in mice. This process depends on the coordinated assembly and disassembly of the actin cytoskeleton which does not occur correctly in global Piezo1 KO mice (35).

## TRPV4

The Transient Receptor Potential (TRP) family of ion channels has many diverse roles, with TRPV4 being the most studied mechanically-gated channel for its role in innate immune cell function (36). TRPV4 is a mechanosensitive, non-selective, Ca^2+^-permeable cation channel that is ubiquitously expressed and responds to both chemical and mechanical signals (37, 38). TRPV4 channel is assembled into a symmetric tetramer with six transmembrane domains, which provide the gating mechanism surrounding a central ion-conducting pore (39). The TRPV4 channel, unlike other channels in the TRP family, lacks an upper gate in its selectivity mechanism, perhaps explaining the relatively nonselective permeability of TRPV4 (39). The exact mechanism of mechanical activation of TRPV4 is unknown, with both direct and indirect mechanisms proposed (38, 40). Similar to Piezo1 activation, mechanical stimuli, such as membrane stretch, may activate the TRPV4 channel, resulting in Ca^2+^ influx (41). Additionally, the TRPV4 channel is regulated by temperature and endogenous ligands, including arachidonic acid metabolites, such as epoxyeicosatrienoic acids (EETs) (42, 43). Conformational change to the cytosolic tail of TRPV4 may increase the likelihood of TRPV4 binding to stimuli-generated messengers (e.g., EET) versus stimulus-generated channel activation (e.g., hypotonicity, heat) (44). Indirect activation occurs through intracellular signaling cascades utilizing focal adhesions, integrins, adhesins, and various second messengers (38). In fact, applying force to β1 integrins resulted in rapid calcium influx through TRPV4 channels (45). These data suggest that TRPV4 is activated by deformation of the cytoskeletal backbone rather than direct membrane stretch (45). Activation of TRPV4, whether direct or indirect, results in Ca^2+^ influx, cytoskeletal rearrangement, intracellular signaling, and altered gene expression which influence cellular phenotype and function (46).

Increased cytosolic Ca^2+^ is necessary for proper phagosome maturation. *M. tuberculosis* can inhibit TRPV4 expression in macrophages, reducing intracellular Ca^2+^ resulting in dysfunctional delivery of mycobacteria to phago-lysosomal components and impaired acidification of phagosomes, which are necessary for effective infection control (47). TRPV4^-/-^ mice had higher mycobacterial colony counts after aerosolization into the lung compared with wild-type mice, and hence impaired control of the initial *M. tuberculosis* infection (24-48 hours after aerosolization) (47). Interestingly, despite worse control of initial infection, TRPV4^-/-^ mice had improved control of chronic infection at 150 days, as shown by reduced colony forming units compared to wild-type mice (47). Improved control of chronic infection was proposed to be secondary to decreased interferon-γ (INF-γ) and diminished neutrophil-driven inflammation in the TRPV4 KO mice (47). *M. tuberculosis* infection results in significant scaring which changes the mechanical properties of the lung. Although TRPV4-dependant control of *M. tuberculosis* in macrophages has not yet been shown to be tissue-stiffness initiated, macrophage function is influenced by a stiffness-dependent mechanism *via* TRPV4 (46). Furthermore, *M. tuberculosis* also induces TRPV4 protein expression in macrophages, suggesting a complex interaction between TRPV4, *M. tuberculosis*, and the mechanical properties of the lung matrix (47). This is a fruitful area for future investigation.

TRPV4 represents an important link between environmental cues and immune cell function (38). Our group has published that TRPV4 in macrophages mediates both LPS-stimulated phagocytosis and downregulation of pro-inflammatory cytokines in both *in vitro* and *in vivo* models (48). The mechanism of TRPV4 action in immune cells has yet to be fully elucidated, but we show that mitogen-activated protein kinases (MAPKs) play an important role (49). We go on to show that TRPV4 regulates phagocytosis and pro-inflammatory cytokine secretion through a molecular switch from JNK to predominately p38 MAPK (49). We further show that the master regulator of MAPK phosphorylation/de-phosphorylation, dual-specificity phosphatase 1 (DUSP1), controls the MAPK molecular switch (49). Overall, our work shows that TRPV4 modulates LPS-induced MAPK switching in stiffness-dependent manner, illustrating the interplay between mechanical force and soluble pro-inflammatory factors that drive innate immunity (49).

TRPV4 activation has also been shown to be instrumental for nuclear translocation of Yes-associated protein/transcriptional co-activator with PDZ-binding motif (YAP/TAZ) in some cell types (50). YAP is a transcriptional co-activator that regulates macrophage polarization towards a pro-inflammatory phenotype in response to substrate stiffness (8, 51). For example, YAP nuclear localization increased with substrate stiffness which correlated with an increase in TNF-α secretion in macrophages plated on substrates of varying stiffness (1, 20, and 280 kPa) (8). The macrophages plated on soft fibrin gel (~0.1 kPa) compared with polystyrene (~10^6^ kPa) had lower total YAP protein, and increased YAP phosphorylation, which leads to its degradation in the cytoplasm (8). The polystyrene-plated macrophages had higher TNF-α secretion in response to LPS, while the fibrin-plated macrophages had increased anti-inflammatory cytokine (IL-10) secretion (8). Therefore, YAP/TAZ participates in the integration of LPS and matrix stiffness signals to modulate macrophage activation. TRPV4 activation has been shown to result in nuclear translocation of YAP/TAZ in other cell types and YAP/TAZ has been shown to mediate macrophage immune function, but whether TRPV4 mediates YAP/TAZ translocation in macrophages in response to pro-inflammatory signals has yet to be shown (8, 51). In summary, TRPV4 signals integrate with canonical inflammatory signals to mediate unique cell type and context-specific responses in macrophages.

## Piezo1 and TRPV4 Crosstalk

**Figure 1:**
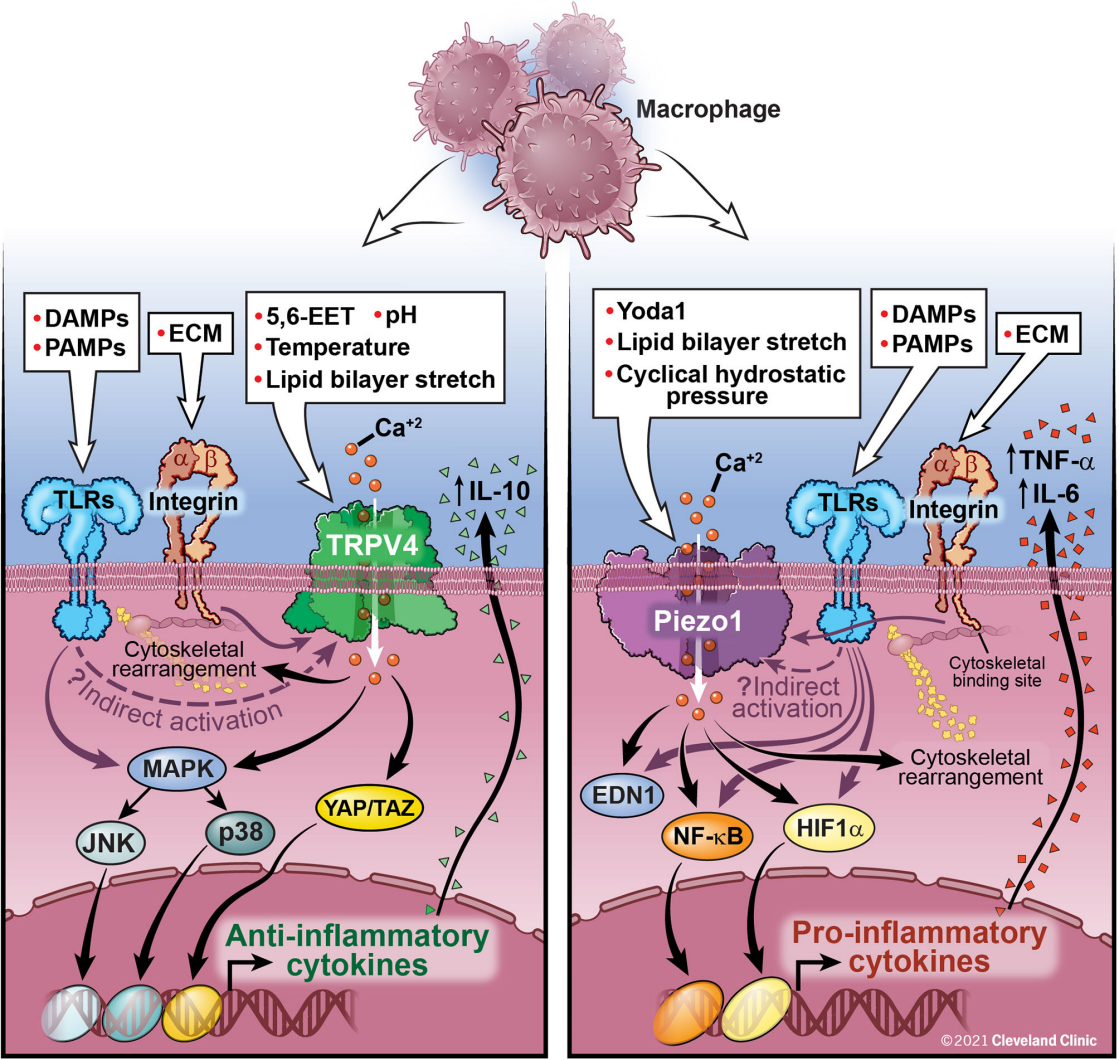
Macrophage activation by TRPV4, Piezo1, and Integrins: TRPV4 is activated by both direct (chemical, mechanical) and indirect mechanisms (downstream signaling through interactions with integrins and TLRs, although the exact mechanism has yet to be elucidated). TRPV4 activation leads to Ca^2+^ influx and activation of several nuclear transcription factors, such as MAPK and YAP/TAZ leading to a decrease in pro-inflammatory cytokines and upregulation of anti-inflammatory cytokines, such as IL-10. Activation of Piezo1, either directly or through interaction with TLRs, leads to Ca^2+^ influx, AP-1 activation, production of EDN1, and nuclear translocation of HIF1α and NF-kB leading to an increase in pro-inflammatory cytokines, IL-6 and TNF-α. Feedback loops between Piezo1 and actin polymerization also contribute to macrophage function. Integrins traverse the cell membrane with an extracellular ligand binding site connecting to an intracellular cytoskeletal bind site. Integrins are important for control of viral and bacterial infections through interactions with TLRs. Further study is needed to fully understand the interactive functions of Piezo1 and TRPV4 and their ability to be activated by integrins and TLRs. These interactions are likely cell-type and context specific.

The possibility of molecular cross-talk between the Piezo1 and TRPV4 channels and its role in cellular responses is an area of active investigation. Not only have Piezo1 and TRPV4 been shown to have individual effects on innate immune function, these channels have also been shown to work in conjunction. While work on TRPV4 and Piezo1 cross-talk has thus far focused on non-immune cells, this work provides insight into possible mechanisms of cooperation between Piezo1 and TRPV4 in immune cells. Based on data from our lab, TRPV4 activation in macrophages by LPS results in anti-inflammatory cytokine secretion (IL-10) and downregulation of pro-inflammatory cytokines, while data from other groups has shown an opposing function of Piezo1 where activation of Piezo1 in a murine *P. aeruginosa* pneumonia model is associated with an increase in pro-inflammatory cytokines (TNF-α, IL-6, IL-1β) (31, 32, 46, 49). We have summarized this data in **Figure 1**. Direct and indirect interactions and cross-talk between Piezo1 and TRPV4 is an area of active investigation.

Piezo1 and TRPV4 have been shown to work synergistically in several pathological states, such as osteoarthritis and pancreatitis. In osteoarthritis, chondrocytes sense physiologic levels of cyclical tensile strain (3% at 0.5 Hz for 8 hours) through TRPV4, while Piezo channels sense excessive, damaging levels of mechanical strain (18% at 0.5 Hz for 8 hours) (52). In pancreatic acinar cells, the activation of Piezo1 by shear stress on pancreatitis acinar cells resulted in a transient elevation of Ca^2+^ that was insufficient to result in the mitochondrial dysfunction and necrosis associated with pancreatitis. Only after activation of TRPV4 by Phospholipase A2 (PLA2) was there a sustained increase in Ca^2+^ which could result in cell death (53). Activation of TRPV4 by PLA2 generated through Piezo1 activation led to a synergistic increase in intracellular Ca^2+^ concentrations with the subsequent pathophysiologic consequences (53). These data demonstrate a complex relationship between Piezo1 and TRPV4. Future work is needed to fully uncover the Piezo1-TRPV4 interactions in inflammatory diseases.

## Adhesion Molecules

Along with ion channels, other molecular families play important roles in mechanotransduction and mechano-responses in immune cells, such as the cytoskeletal proteins, actin and myosin, focal adhesions, selectins, and integrins. Due to the focused nature of this review, we direct you to more comprehensive reviews in this area (15, 54–56). The cytoskeletal-extracellular matrix interactions have an important role in mechanotransduction by enhancing intracellular signals, activating surface receptors, triggering migration, and cell-to-cell communication in macrophages and neutrophils. The cytoskeletal-extracellular matrix interaction is also necessary for cells to exert force (15). The generation of force is critical for several immune cell functions, such as leukocyte extravasation. Leukocytic integrins connect endothelial cell ligands to intracellular actin and myosin filaments through focal adhesions which serve as anchors to help leukocytes overcome vascular flow in order to initiate adhesion and rolling (15, 56, 57). However, looser associations between selectins and their ligands allow leukocytes to roll along the endothelial surface. These loose associations are characterized as “slip bonds,” which weaken under increased tension (58). E-selectin ligands (ESL) allow neutrophils to transduce mechanical signals regarding blood flow and shear force that facilitate the slow rolling needed for effective immunosurveillance (59).

Integrins are transmembrane, heterodimeric proteins, with α and β subunits, which connect the extracellular matrix with the cytoskeleton and mediate intracellular signaling (54, 60). The tensegrity model suggests that integrins act as mechanotranducers by mediating force-induced rearrangements in the cytoskeleton (61). During leukocyte extravasation, chemokines and bacterial surface molecules (e.g. LPS), promote expression of integrins which strengthen the adhesion of leukocytes through the creation of “catch bonds” (60, 62). Catch bonds, unlike slip bonds, become stronger under tension until an optimal tension is exceeded. Cytoskeletal rearrangements mediated through integrins are necessary for the creation of pseudopodia which project between endothelial cells prior to transmigration for effective leukocyte recruitment to site of infection (63). In contrast, the first step of neutrophil extravasation in the lung is dependent on cytoskeletal rearrangements into submembrane F-actin rims in the neutrophil, resulting in neutrophil sequestration in response to lung infection (64).

Given the known interaction between TRPV4, integrins, and toll-like receptors (TLRs), it is worthy to mention the importance of epithelial/endothelial barrier function in the host response to infection. For example, integrin αvβ3 on epithelial/endothelial cells has been shown to be integral to the containment of herpes simplex virus (HSV). Integrin αvβ3 directs HSV to the acidic endosome pathway by re-localizing nectin-1, a cellular protein which mediates the entry of HSV (65). Integrin αvβ3 interactions with toll-like receptor 2 (TLR 2) and viral proteins lead to production of type 1 interferons and NF-kB which helps suppress HSV replication. These functions suggest αvβ3-integrin has an important role in mediating intracellular uptake of HSV and activation of innate immune signaling response (66).

Phagocytosis, the process by which a cell engulfs extracellular particles/pathogens *via* cytoskeletal rearrangement, is essential to immune cell function. Upon activation of TLRs and G-protein coupled receptors (GPCR) by chemokines and LPS, phagocytes exit their resting state and undergo actin remodeling to become primed for interaction with phagocytic targets (55). Priming of phagocytes leads to the expression of phagocytotic receptors and growth of the phagocytotic cup (55). Similar to lamellipodial growth, expansion of the phagocytotic cup occurs by accumulation and arrangement of F-actin into a peripheral ring (15). Furthermore, during phagocytosis catch bonds form between the macrophage filopodia glycoprotein CD48 and the adhesin FimH on the fimbrae of *Escherichia coli*. This stable connection triggers formation of a lamellipodium to scoop up the surface bound pathogens and shovel them into the phagocytotic cup (67). Once the pathogen is internalized, the phagosome undergoes maturation, preparing for pathogen killing and disposal. Fusion of the phagosome with proteolytic enzyme-rich granules and acidic lysosomes facilitates pathogen killing (68).

While the mechanism is not fully understood, the observation that constraints on macrophage cell shape induce changes to macrophage immune function suggests the important role of the cytoskeleton in determining macrophage inflammatory phenotype (69). This occurs through a dynamic bidirectional interaction, in which both outside-in signals (mechanical, physical, temperature, and chemical) and inside-out cytoskeletal modifications can affect macrophage function. Our lab has shown that matrix-stiffness is a critical factor in determining the effectiveness of phagocytosis and pathogen clearance, which is blocked in the absence of TRPV4 (46, 49). Improved overall pathogen clearance mediated by TRPV4 in the presence a stiff matrix is depends in part on cytoskeletal remodeling. That said, other processes including neutrophil recruitment and degranulation, reactive oxygen species generation, myeloperoxidase production, and cytokine and resolvin secretion contribute to overall pathogen clearance and resolution of infection.

## Clinical Implications

Mechanotransduction in innate immune cells has been shown to have an impact on multiple disease states, including non-infectious (e.g. ventilator-associated and hydrochloric acid [HCl]) and infectious (e.g. pneumonia) inducers of lung injury (48, 70). In non-infectious inflammation, rapid influx of Ca^2+^ *via* TRPV4 in murine pulmonary endothelial cells undergoing mechanical ventilation with high peak inspiratory pressures (25 and 35 cmH_2_O) leads to increased permeability, rapid accumulation of inflammatory cell infiltrates, and pulmonary edema (71, 72). These changes in surface tension on the alveolar surface can influence macrophage phagocytotic activity, with increasing alveolar surface tension associated with a reduction in effective macrophage phagocytosis (73). In a murine model of acute respiratory distress syndrome secondary to instillation of hydrochloric acid (HCl), mimicking aspiration-induced lung injury, TRPV4 KO lungs perfused with TRPV4^+/+^ leukocytes had increased neutrophil activation, respiratory burst, and neutrophil adhesion and migration compared to TRPV4 KO lungs perfused with blood from TRPV4 KOs, suggesting that neutrophil TRPV4 mediates the acute cellular inflammatory response. On the other hand, vascular leak and histologic signs of lung injury were mediated by endothelial TRPV4, rather than neutrophil TRPV4 activation (70). Pharmacologic inhibition of TRPV4 attenuated the sequelae of acute lung injury secondary to HCl exposure, including the breakdown of the endothelial barrier, lung inflammation, and histologic signs of lung injury. This attenuation of lung injury occurred only if the TRPV4 inhibitor was given prior to the administration of HCl (70). On the other hand, post-exposure pharmacologic inhibition of TRPV4 suppressed pulmonary inflammation from chemically-induced lung injury through reductions in macrophages, neutrophils, and pro-inflammatory cytokines when given 30 minutes after HCl or chlorine gas administration (74).

In regards to infection-associated inflammation, TRPV4 has been noted to be activated by heat which may provide a mechanism for immune cell activation in response to changes in body temperature (43, 75). Febrile-range temperatures are associated with more effective recruitment and respiratory burst in neutrophils and increased bacterial clearance and cytokine secretion in macrophages (76). As previously discussed, both Piezo1 and TRPV4 activation leads to increased bacterial clearance. During cyclical hydrostatic pressure conditions, mice with absent Piezo1 in macrophages were shown to be unable to control infection after intranasal *P. aeruginosa* (31). Similarly, our work showed increased clearance of intrapulmonary *P. aeruginosa* in wild-type mice compared to TRPV4 KOs in a stiffness-dependent fashion (48). However, in a murine model of sepsis, pharmacologic inhibition of TRPV4 improved survival and reduced pro-inflammatory cytokines, including TNF-α and IL-6 (77). These studies demonstrate a context and model-system specificity and suggest that the role of TRPV4 in infection and inflammation remains to be fully understood.

Understanding the mechanisms by which immune cells sense and respond to mechanosensitive tissue signals presents potential targets to treat human disease. Limited clinical trials have been performed using pharmacologic inhibition of TRPV4 by GSK2798745 (GSK; GlaxoSmithKline, London, UK) (78). The safety and tolerability of the pharmacologic inhibitor of TRPV4, GSK, has been demonstrated in both healthy human subjects and in those with compensated heart failure (79). There have been clinical trials evaluating the potential therapeutic benefit of GSK for several conditions including: cardiogenic pulmonary edema, chronic cough, and LPS-induced lung injury, although the trials for lung injury and chronic cough were terminated early because they were unlikely to reach their primary end-points (80–82). The scientific basis for these trials is to inhibit TRPV4’s role in vasodilation and vascular permeability, which is likely mediated by both endothelial and immune cell dysfunction (83). TRPV4 inhibition has even been proposed as a possible therapeutic agent preventing damage to the alveolar-capillary barrier associated with Coronavirus Disease 2019 (COVID-19) (84).

## Future Directions

Ongoing study of mechanobiology in innate immune cells offers further understanding of human disease and opportunities for potential therapeutics. Future work is needed to target mechanosensitive channels or adhesion molecules to control intracellular signal transduction and modulate disease, with particular emphasis on examining Piezo1 and TRPV4 interactions during inflammation. While clinical trials in humans have thus far failed to show efficacy, opportunities to target specific cell types or interacting partners may yield greater efficacy. Future therapeutic targets may be identified from further understanding of downstream signaling pathways, with important areas of future study including autoimmune, allergic, infectious, and malignant conditions. Research has only begun to elucidate the mechanisms involved in innate immune cell mechanotransduction and their potential involvement in a variety of human diseases leads to vast opportunities for ongoing study.

## Author Contributions

EMO, MAO, and RGS contributed to the conception and writing of the manuscript. AP, BDS, and LMG provided critical revisions to the article. All authors contributed to the article and approved the submitted version.

## Funding

This work was supported by NIH grants (HL132079) to BDS, (R01HL-133721 and R01HL-158746) to MAO, and (K08HL-133380 and R01HL-155064) to RGS, the Ann Theodore Award to MAO and RGS, and the SMARRT T32 which is funded by the National Institutes of Heart, Lung, and Blood grant, T32HL-155005. The content is solely the responsibility of the authors and does not necessarily represent the official views of the NIH. 

## Conflict of Interest

The authors declare that the research was conducted in the absence of any commercial or financial relationships that could be construed as a potential conflict of interest.

## Publisher’s Note

All claims expressed in this article are solely those of the authors and do not necessarily represent those of their affiliated organizations, or those of the publisher, the editors and the reviewers. Any product that may be evaluated in this article, or claim that may be made by its manufacturer, is not guaranteed or endorsed by the publisher.
